# Coding Anisotropic Metasurface with Integrated Broadband Tunable Radiation and Low-Scattering Performance

**DOI:** 10.1186/s11671-019-2944-8

**Published:** 2019-03-29

**Authors:** Li Li Cong, Xiang Yu Cao, Huanhuan Yang, Jun Gao, Tao Song

**Affiliations:** Air Force Engineering University, Xi’an, 710077 China

**Keywords:** Coding metasurface, Diffusion, Radar cross section, Polarization reconfigurable, Array antenna

## Abstract

In this paper, we propose a coding electromagnetic metasurface (EMMS) with integrated broadband tunable radiation and low-scattering performance. Anisotropic elements demonstrating opposite phases under *x*- and *y*-polarized incidence are investigated and coded as “0” and “1” basic elements. These elements are then arranged in an optimized layout using a simulated annealing algorithm to perform the EMMS. By this means, diffusion scattering is realized in a broadband. Meanwhile, when “0” and “1” are fed properly, the coding EMMS displays wideband linearly or circularly polarized radiation with symmetric profiles. Simulated and experimental results verify that our method offers a simple and ingenious way to integrate broadband radiation and low scattering into one single-coding EMMS.

## Background

Electromagnetic (EM) metasurfaces (EMMSs), artificially constructed by periodic or quasi-periodic sub-wavelength particles, are denoted as a surface version of three-dimensional metamaterials [1, 2]. By virtue of compact structures, low profile, good conformal shape, low cost, and easy fabrication, the EMMSs have been extensively investigated and engineered to manipulate EM waves [3–9], such as polarization, amplitude, and phase.

Especially, anisotropic EMMSs are more ready to achieve a number of interesting characteristics not possible with isotropic ones in some occasions. For polarization engineering, by employing anisotropic particles to construct reflective or transmissive polarization conversion EMMSs, one can almost realize arbitrary polarizations from one specific polarization, such as linear polarization to linear polarization [10–13], linear polarization to circular polarization [14–16], circular polarization to circular polarization [17, 18], and so on. Circularly polarized antennas, polarization-controlling devices, and radar cross section reduction (RCSR) can be further accomplished based on polarization manipulation. Absorption is a common fashion for amplitude manipulation. Through changing relative gap orientations or neighboring center offsets of multilayered anisotropic split-ring resonators [19–21], one can tune the near-field interactions between them. By this means, low reflection and transmission can be simultaneously obtained to achieve perfect absorption. As for phase manipulation, by delicately designing the geometry of sub-wavelength particles of the EMMS, phase discontinuities imparted across the reflected or transmitted surface can be achieved. Thus, many fascinating EM devices, such as metasurface lens [22, 23], metasurface holograms [24, 25], invisible cloaking [6], spin-orbit manipulation [26, 27], and some other functional interfaces [28–31], can be then realized.

Recently, coding EMMSs have gained intensive attention as another paradigm for manipulating EM wave propagation [32–35]. The “coded bits” are represented by constitutive particles with different phase responses. Take 1-bit EMMS as an example, coded “0” and “1” elements are mimicked by constitutive structures with 0° and 180° phase shift, respectively. Through a certain spatial mixture of these coded elements, 2-bit, 3-bit, and multi-bit EMMSs can be subsequently accomplished [36–38]. With multifunction and tunability demands of EM devices, switchable components and field-programmable gate array hardware are included in coding EMMS design. Hence, reconfigurable [39] and programmable [40] EMMSs are then obtained. Based on the aforementioned “coding” concept, 0-bit EMMS, consisting of only one kind of anisotropic elements, can be used to achieve polarization conversion [39], while multi-bit EMMSs coded by optimization algorithms can be used to manipulate diffusion scattering performance, thus achieving RCSR [39].

Obviously, abovementioned EMMS designs mainly devote to investigate scattering performance for incoming EM wave. Actually, if fed appropriately, the EMMSs themselves can act as antennas to radiate EM wave [41–46]. Furthermore, to the best of the authors’ knowledge, the “coding” concepts mainly focus on scattering evaluation, but not included in radiation performance. In this paper, the proposed EMMS involves broadband radiation and low-scattering performance simultaneously. The EMMS is composed of anisotropic elements, which possesses opposite phases under *x*- and *y*-polarized incidence. These anisotropic elements are coded as “0” and “1” and then arranged in a certain sequence optimized by the simulated annealing algorithm (SAA). Based on the antenna array theory [47], appropriate feeding structures are added to coding “0” and “1” elements to realize desired radiation performance. If “0” and “1” elements are fed with the same amplitude and phase, linearly polarized (LP) radiation can be achieved. While if “0” and “1” elements are fed with the same amplitude but with 90° phase difference, left- or right-hand circular polarization (L/RHCP) radiation can be achieved. Meanwhile, the optimized layout of EMMS results in broadband diffusion scattering performance for incoming EM wave, which is to the advantage of bistatic RCSR. Both simulation and measurement prove that our method offers a simple, flexible, and ingenious strategy for EMMS design with integrated broadband radiation and low scattering performance.

## Methods

**Fig. 1:**
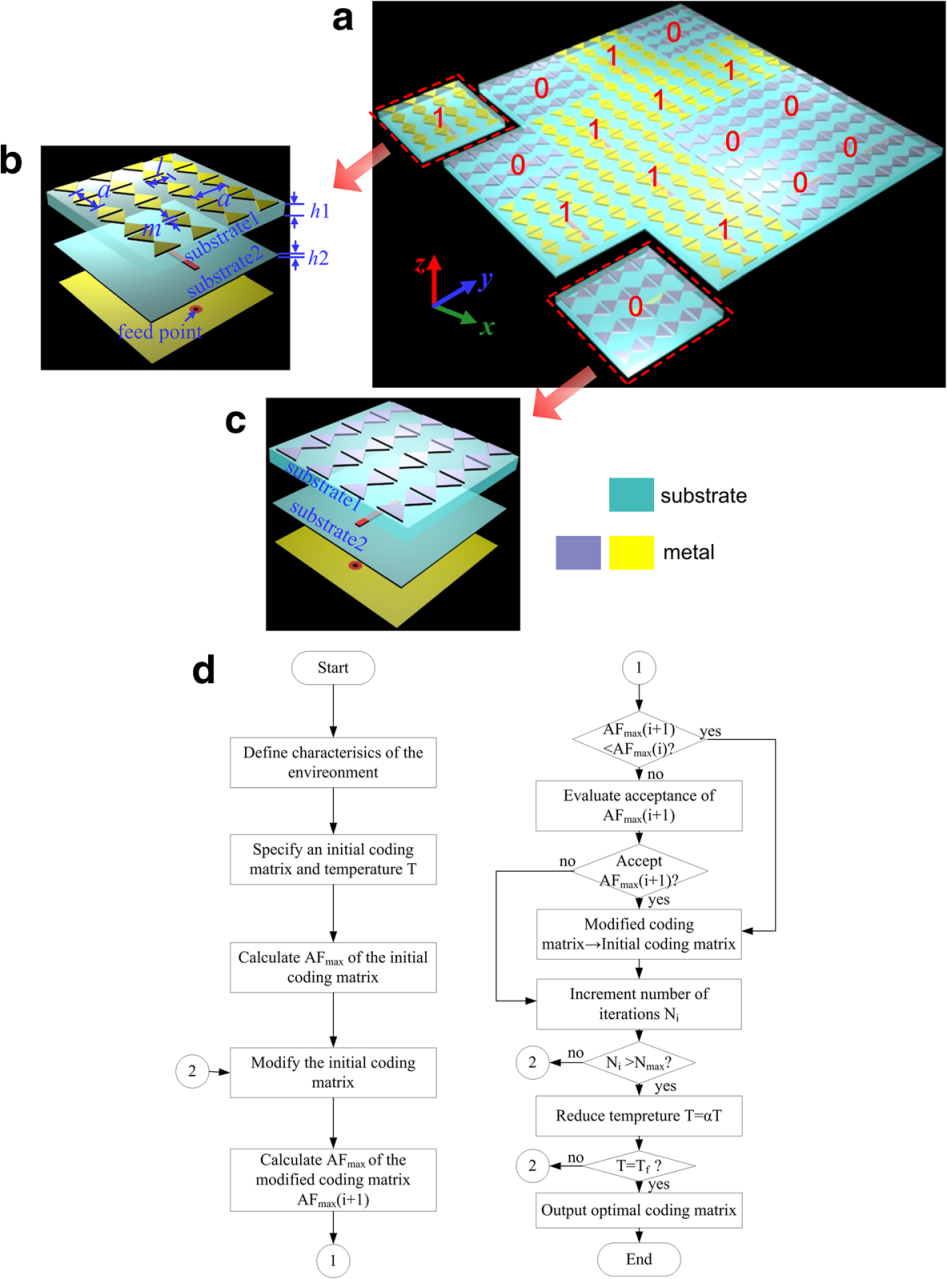
Coding EMMS and its constituent anisotropic element. **a** Coding EMMS consists of 4 × 4 pieces of anisotropic elements. The numbers of “0” and “1” elements are the same. Schematic geometry of the anisotropic “1” element (**b**) and “0” element (**c**) (*a* = 9 mm, *l* = 6 mm, *m* = 1 mm, *h*1 = 3 mm, *h*2 = 0.5 mm). **d** Flowchart of the SAA for finding the optimal coding matrix

Figure 1 depicts the detailed geometry of the coding EMMS and the constitutive anisotropic elements. Two FR2 dielectric layers (dielectric constant of 2.65, loss tangent of 0.002) are employed as substrates, denoted as substrate1 and substrate2. The two dielectric layers are tightly and flatly stacked together without any air space between them. The thicknesses of the substrates from top to bottom are 3 mm and 0.5 mm, respectively. 4 × 4 bowtie-shaped metallic patches are etched on the top surface of substrate1 measuring 36 × 36 mm^2^ (equal to 0.66*λ*_0_ × 0.66*λ*_0_ at 5.5 GHz). The metallic ground plate with a slot as thin as possible (length of 15.5 mm, width of 0.2 mm) is etched on the bottom surface of substrate2 to ensure absolute reflection. Apparently, the EM properties of such anisotropic element lie in its physical arrangement. Based on the “coding” concept, the anisotropic element shown in Fig. 1b is nominated as “1” , while its counterpart (90°rotation around *z*-axis) is denoted as “0”. The layout of finally proposed EMMS is optimized by SAA, which is a method for local searching. Figure 1d shows the flow chart of the SAA for achieving the optimal coding matrix. It begins with an initial solution which is randomly modified in an iterative process. The main parameters of SAA involve the initial temperature *T*, the decreasing rate *α* of each iteration process, the final temperature *Tf*, the number of iterations *I*, and the merit function. In our model, we define an initial coding matrix with equal number of “0” and “1”. It is then upgraded by changing positions of an arbitrary pair of “0”and “1”. The parameters *T*, *α*, *Tf*, and *I* are set as 100, 0.9, 0, and 1000, respectively. For low RCS performance, good diffusion scattering is expected. Thus, our goal is to find the optimal coding matrix (*M*_best_) leading to a desired scattering pattern with the smallest maximum value. Thus, the issue is a min-max problem in which the merit function can be expressed as *F*(*M*_best_) = min(AF_max_), where AF_max_ is the maximum value of AF corresponding to a given coding matrix. The optimal coding matrix corresponds to the minimum AF_max_, which would lead to a perfect diffusion scattering performance. Generally, the bigger the array size is, the better diffusion scattering we obtain. Here, we choose an array consisting of 4 × 4 elements (*M* = *N* = 4). Finally, the optimal coding matrix is shown in Fig. 1a. All simulations in the following analysis unless otherwise stated are carried out with aid of the commercial simulation software Ansoft HFSS v.14.0.

**Fig. 2:**
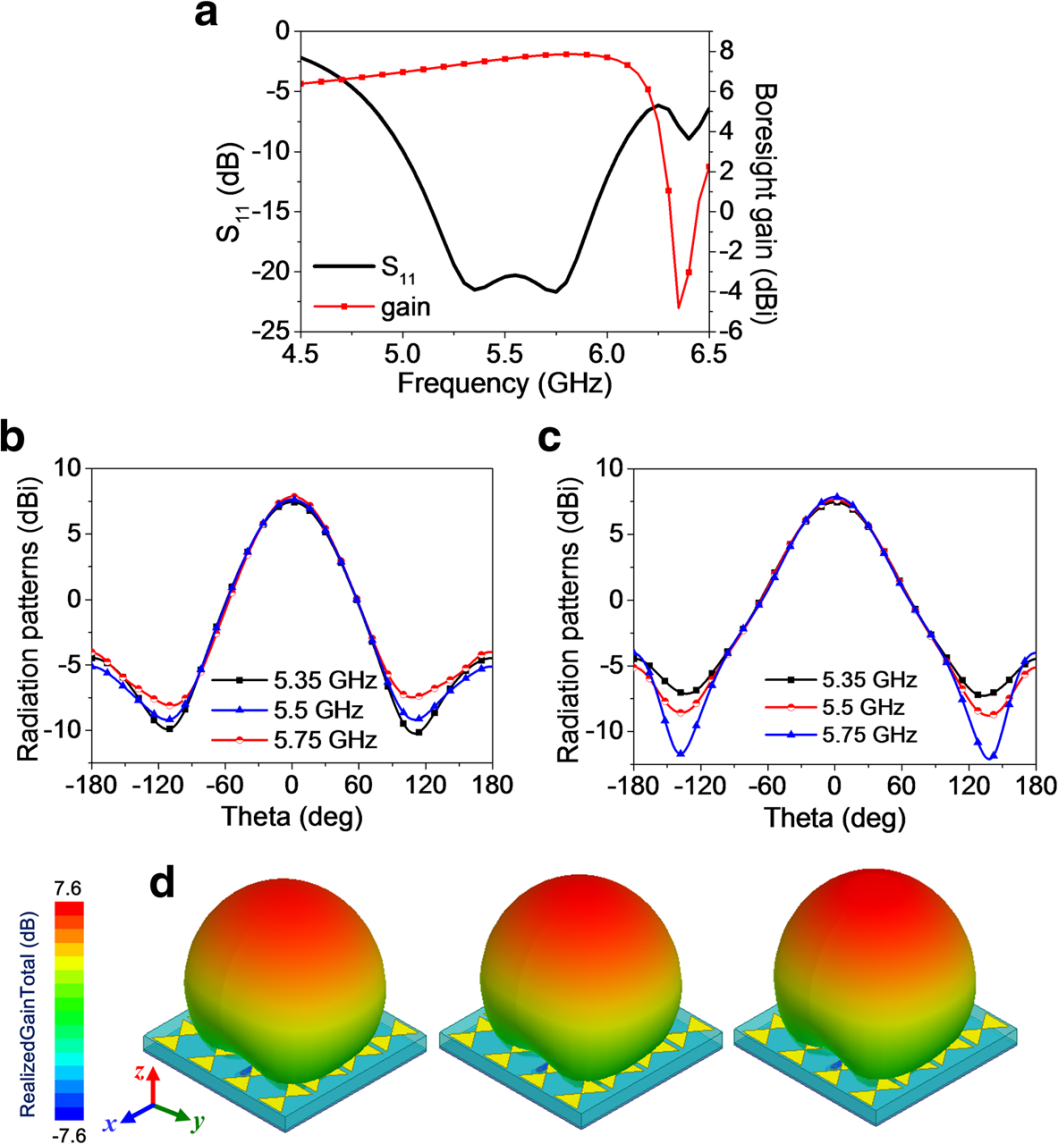
Radiation properties of the anisotropic element with lumped port excitation. **a** Reflection coefficient S_11_ and boresight gain versus frequency. 2D radiation patterns at **b** xoz- (E-) and **c** yoz- (H-) plane. **d** 3D radiation patterns at 5.35, 5.5, and 5.75 GHz (from left to right)

For the radiation case, lumped port excitation and radiation boundary are applied on the anisotropic element. A 50-Ω SMA is connected to the extremely thin rectangular patch (length of 13 mm, width of 1.3 mm) through a small hole in substrate2 for impedance matching. The slot in the metallic ground then takes effect by coupling energy to the top anisotropic EMMS to radiate LP EM wave. The reflection coefficient S_11_ and radiation patterns are plotted in Fig. 2. As clearly observed, the bandwidth for − 10 dB impedance matching is achieved from 5 GHz to 6 GHz, implying a relative bandwidth of 18.2%. A stable boresight gain varying from 6.97 dBi to 7.86 dBi is obtained over the impedance bandwidth. Meanwhile, a normal and symmetric radiation profiles are observed in broadside direction for both xoz- (E-) and yoz- (H-) planes, as clearly shown in Fig. 2b–d.

**Fig. 3:**
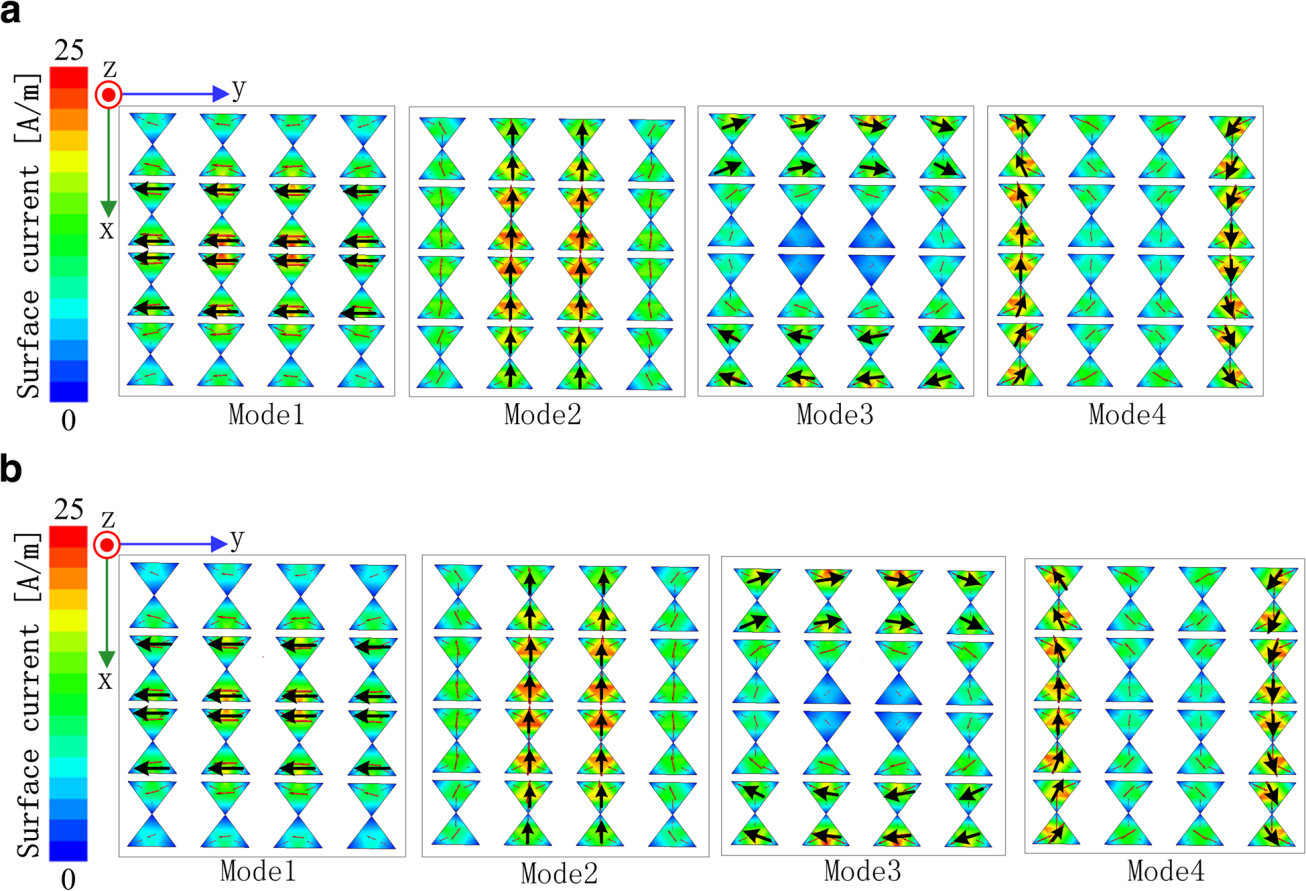
Modal surface current of mode 1, mode 2, mode 3 and mode 4. **a** 5.35 GHz and **b** 5.75 GHz

**Fig. 4:**
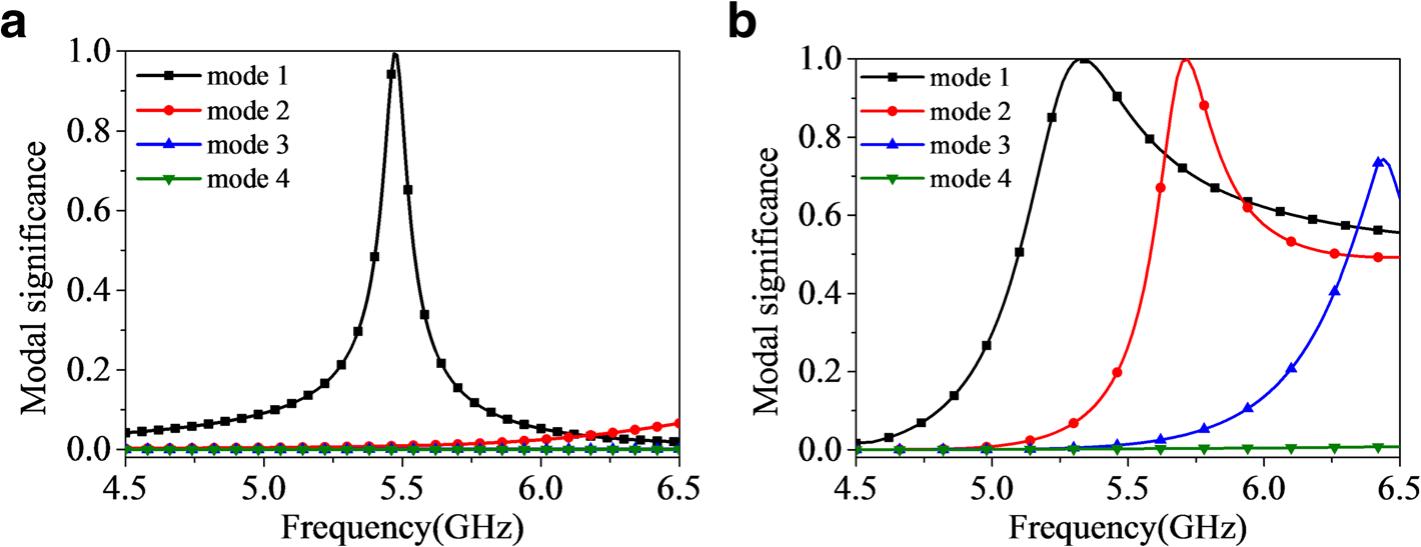
Modal significance of the anisotropic element with (**a**) and without (**b**) bowtie-shaped metasurface

To give a physical insight into the working mechanism, the modal surface current of the anisotropic element at 5.35 GHz and 5.75 GHz is plotted in Fig. 3a and b. Note that the simulations performed in this section were carried out by using FEKO 7.0. As clearly shown, the surface current of mode 1 and mode 2 mainly distributes on the middle patches which may result in broadside radiation, while that of unwanted mode 3 and mode 4 mainly distributes on the edge patches, which may result in radiation nulls in the broadside. Furthermore, the surface current of mode 1 and mode 3 flows along the *y*-axis, while that of mode 2 and mode 4 flows along the *x*-axis. Besides, the calculated modal significances of the first four characteristic modes of the anisotropic element with and without metasurface are illustrated in Fig. 4a and b. We can tell from Fig. 4b that when the metasurface is applied on the element, mode 1 and mode 2 are resonant at 5.32 GHz and 5.72 GHz in the desired operation band, with either of their modal significances approaching unity. Thus, mode 1 and mode 2 are the fundamental orthogonal mode pairs to generate the wideband and broadside radiation patterns.

**Fig. 5:**
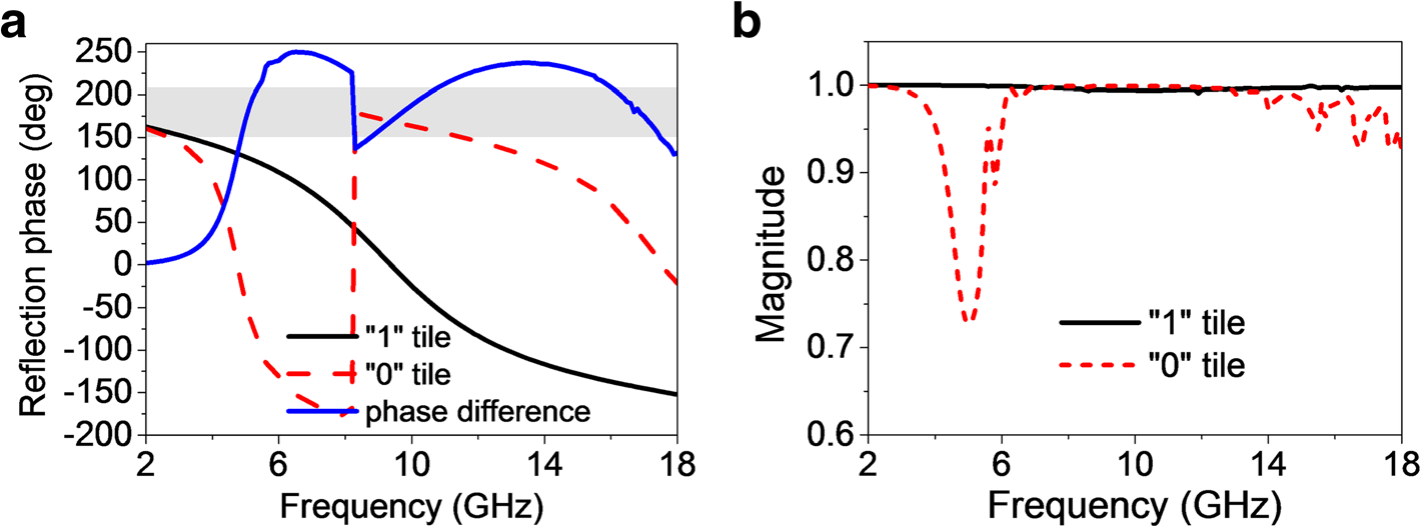
Reflection characteristics of the anisotropic element with floquet port excitation. **a** Reflection phases and phase difference between “0” and “1” elements. **b** Reflection magnitudes

For the scattering case, floquet port excitation and master/slave boundaries are implemented on the anisotropic element to exploit the reflection characteristics. As plotted in Fig. 5, only one 0° reflection phase point arises at 9.38 GHz for “1” element, while dual 0° reflection phase points appear at 4.75 GHz and 17.52 GHz for “0” element. Thus, an effective reflection phase difference is created between “0” and “1” elements, as indicated in the dark grey part in Fig. 5a. Meanwhile, the reflection magnitudes shown in Fig. 5b maintain close to 1 in 2~18 GHz for both elements. It is worth noting that a hollow zone for reflection magnitude response is observed around the working band (5~6 GHz) of “0” element. This is attributed that part of the co-polarized energy is absorbed by the feeding structure. Still and all, energy cancellation [47] can be well obtained in a broadband. Consequently, broadband RCSR can be expected.

## Results and Discussion

In some sense, the scattering process can be understood by transforming EM wave reflection to re-radiation process. Therefore, for an *M* × *N* EMMS array, the working principle for both radiation and scattering cases can be interpreted by the standard array theory [47]:

1 $$ {E}_{\mathrm{total}}=\mathrm{EP}\cdot AF=\sum \limits_{m=0}^{M-1}\sum \limits_{n=0}^{N-1}{\mathrm{EP}}_{\left(m,n\right)}\cdot {e}^{j\left[ km\Delta x\sin \theta \cos \varphi + kn\Delta y\sin \theta \sin \varphi +\phi \left(m,n\right)\right]} $$

where EP is the pattern function of a single element, *AF* is the array factor, *k* is the wavenumber, Δ*x* and Δ*y* are the distance between adjacent elements along *x*- and *y*-directions, respectively, *ϕ*(*m*, *n*) is the phase of the (*m*, *n*) element, and *θ* and *φ* are the elevation and azimuth angle of an incidence. For simplicity, the subscripts of *E*_rtotal_ and *E*_stotal_ in the following analysis indicate cases of radiation and scattering, respectively.

For the radiation case, all anisotropic elements act as radiators when fed appropriately. Naturally, the “0” and “1” elements would produce two orthogonally polarized electric fields, namely EP_'0'_ ⊥ EP_'1'_. Then, the polarization of radiated EM wave from EMMS depends on the amplitude and phase of the feed sources. Assuming that the input power of each element is equal, one would have |EP_'0'_| = |EP_'1'_|. *ϕ*(*m*, *n*) would represent the input phase from feed sources. Hence, along the normal direction with (*θ*, *φ*) = (0^∘^, 0^∘^), Eq. (1) would be simplified as ${E}_{\mathrm{rtotal}}=8\left({\mathrm{EP}}_{\hbox{'}0\hbox{'}}{e}^{j{\phi}_{\hbox{'}0\hbox{'}}}+{\mathrm{EP}}_{\hbox{'}1\hbox{'}}{e}^{j{\phi}_{\hbox{'}1\hbox{'}}}\right)$ for the proposed EMMS. If *ϕ*_'0'_ − *ϕ*_'1'_ = 0^°^or ± 180^°^, the total radiation would be LP within the diagonal planes. If *ϕ*_'0'_ is 90°ahead of *ϕ*_'1'_, the total radiated field would be RHCP. Otherwise, if *ϕ*_'0'_ falls 90°behind *ϕ*_'1'_, LHCP radiation would be generated. To summarize, the polarization of radiated field from the EMMS can be adjusted at will by controlling input phases of “0” and “1” elements.

**Fig. 6:**
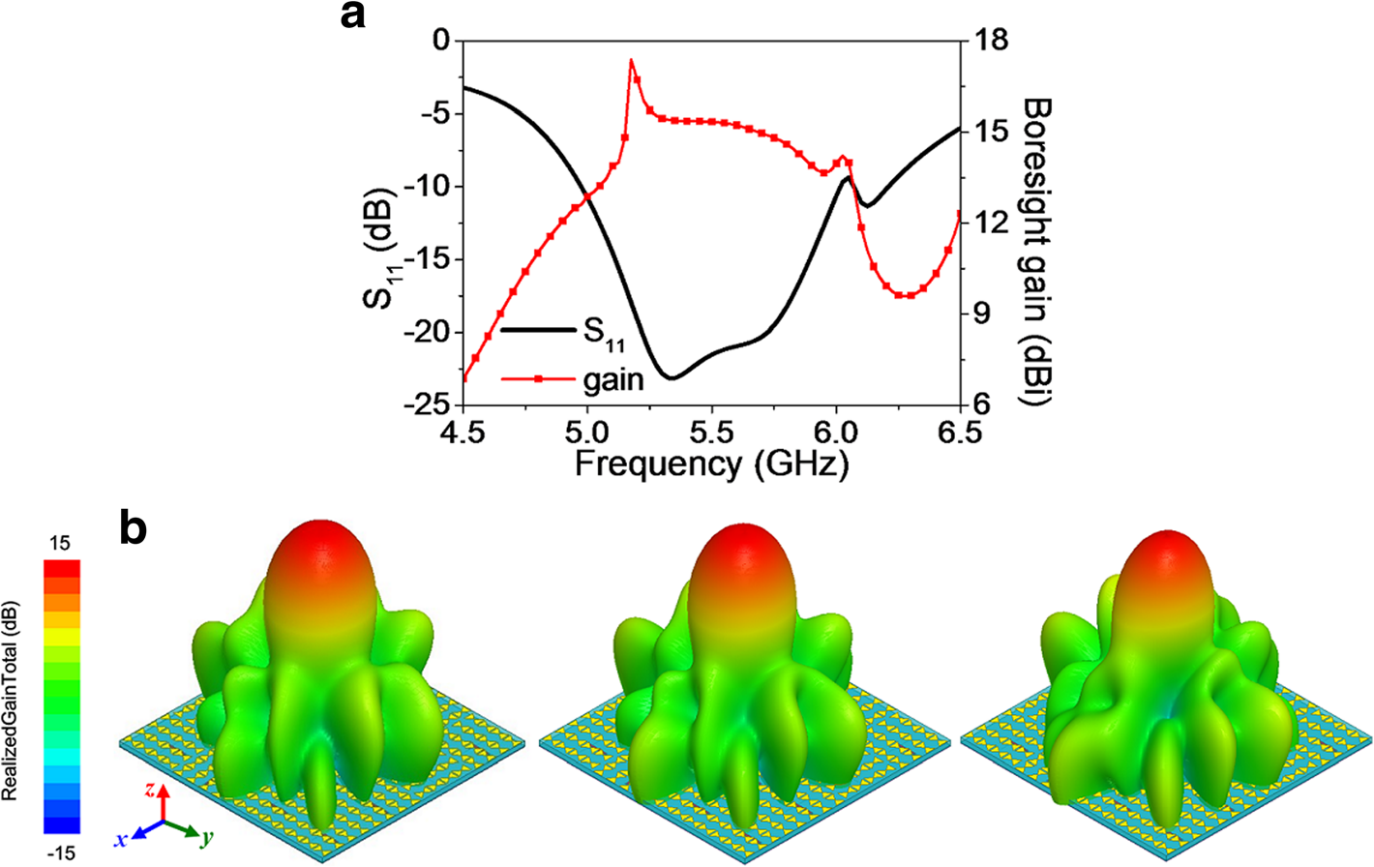
Linear radiation properties of the EMMS with “0” and “1” fed with equal magnitude and phase. **a** Reflection coefficient S_11_ and boresight gain versus frequency. **b** 3D LP radiation patterns at 5.35, 5.5, and 5.75 GHz (from left to right)

**Fig. 7:**
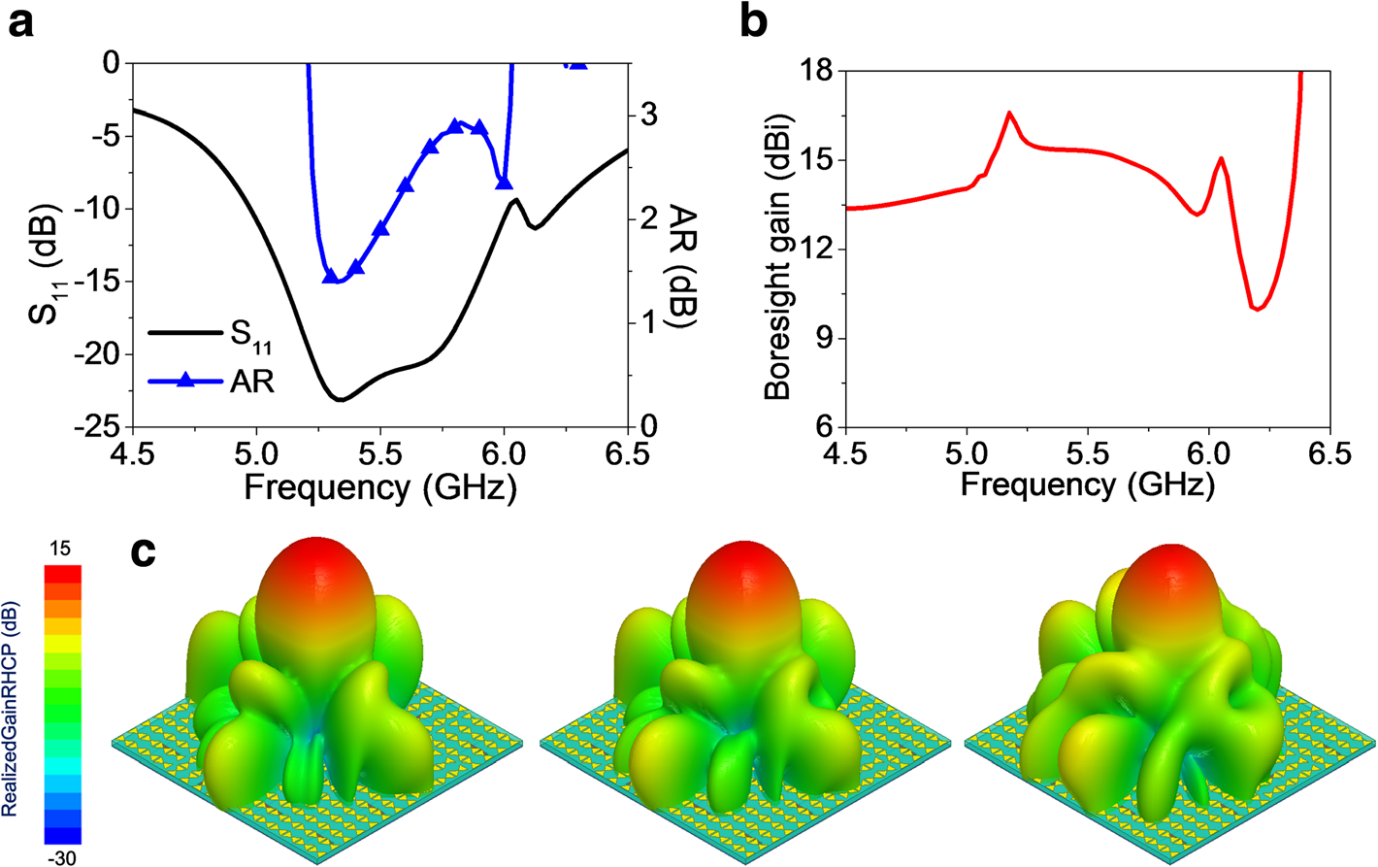
RHCP radiation properties of the EMMS with “0” and “1” fed with equal magnitude and 90° phase shift. **a** S_11_ and AR versus frequency. **b** Boresight gain versus frequency. **c** 3D RHCP radiation patterns at 5.35, 5.5, and 5.75 GHz (from left to right)

For briefness of the paper, only two representative cases are involved in the following analysis. All “0” and “1” elements are fed with equal power in both cases. On one hand, in terms of *ϕ*_'0'_ = *ϕ*_'1'_ = 0^°^, LP radiation performances are obtained as depicted in Fig. 6. Good impedance matching is achieved from 4.97 GHz to 6.05 GHz (19.6% relative bandwidth), while the gain in normal direction varies from 12.6 dBi to 17.38 dBi in the operation band. Symmetrical radiation patterns are observed in the broadside direction for both E- and H-planes, as clearly shown in Fig. 6b. On the other hand, when *ϕ*_'1'_ − *ϕ*_'0'_ = 90^°^, RHCP radiation is observed as expected. As shown in Fig. 7, the bandwidth for S_11_ < − 10 dB and 3 dB axial ratio bandwidth (ARBW) is 4.97~6 GHz and 5.22~6 GHz, respectively. The common bandwidth for S_11_ < − 10 dB and 3 dB ARBW is from 5.22 GHz to 6 GHz (13.9% relative bandwidth), with boresight gain varying from 13.16 dBi to 15.8 dBi. Likewise, symmetric, broadside, and normal radiation profiles are observed in the 3D radiation patterns at 5.35, 5.5, and 5.75 GHz.

**Fig. 8:**
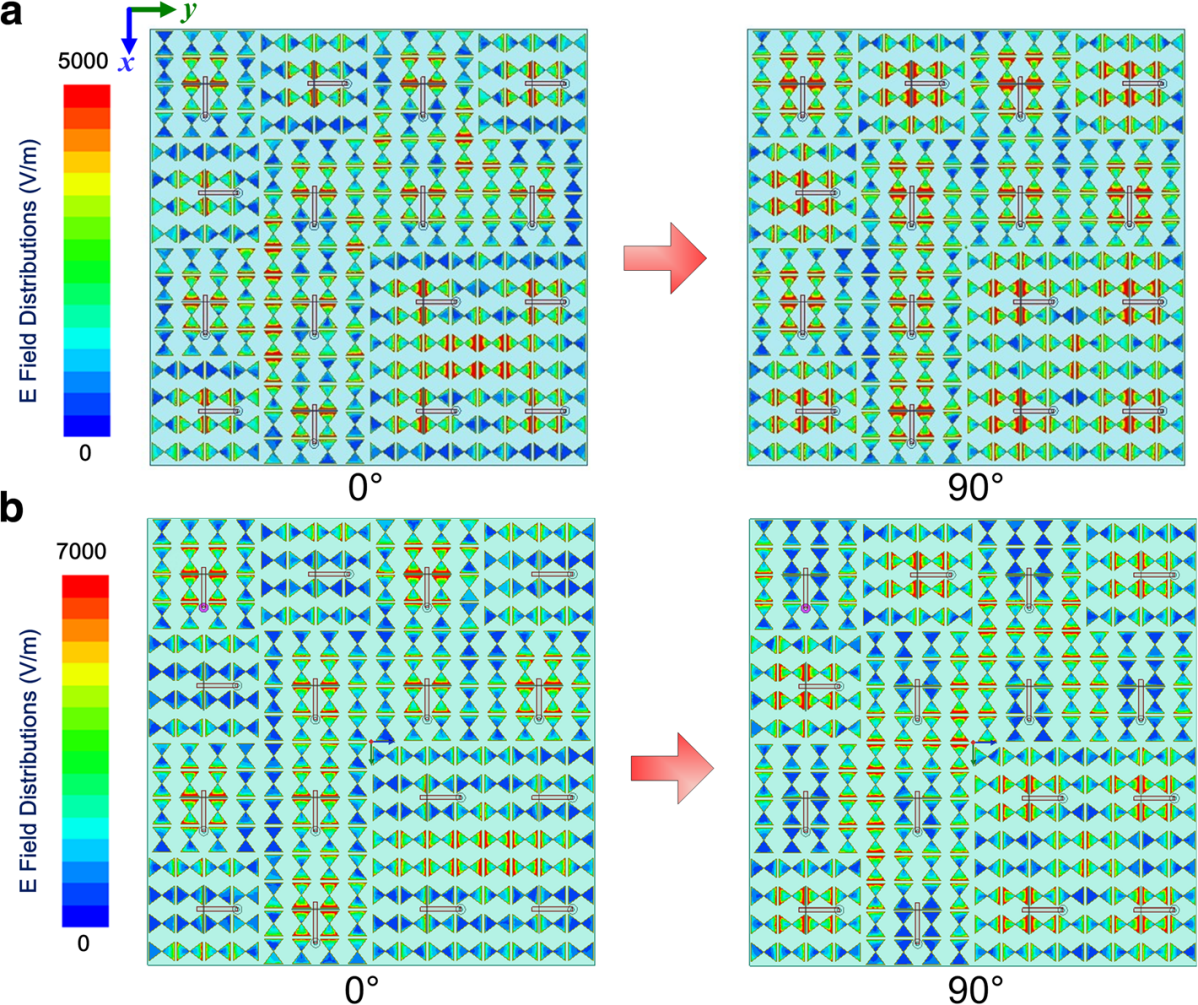
Electric field distributions of the EMMS at 5.35 GHz with different time variants. **a** LP radiation case. **b** RHCP radiation case

From the aforementioned analysis, it can be verified that the proposed EMMS can perform as a good antenna and radiate in linear polarization and circular polarization modes alternatively by controlling the input magnitudes and phases. Meanwhile, the simulated results indicate that the working bandwidth of proposed EMMS maintains well compared with a single anisotropic element, which verifies the effectiveness of our proposed method. To get an intuitive insight into the working mechanisms of the EMMS for different radiation modes, the electric field distributions at 5.35 GHz with different time variants are investigated. It is clearly shown in Fig. 8a that the resonant E-field distributes evenly across “0” and “1” elements all along as time changes for LP radiation. However, for CP radiation, “1” elements exhibit stronger field density at the phase of 0°, while “0” elements prevail over “1” ones at the phase of 90°. Thus, two orthogonal modes with a 0°or 90° phase difference are excited to perform LP or CP radiation.

**Fig. 9:**
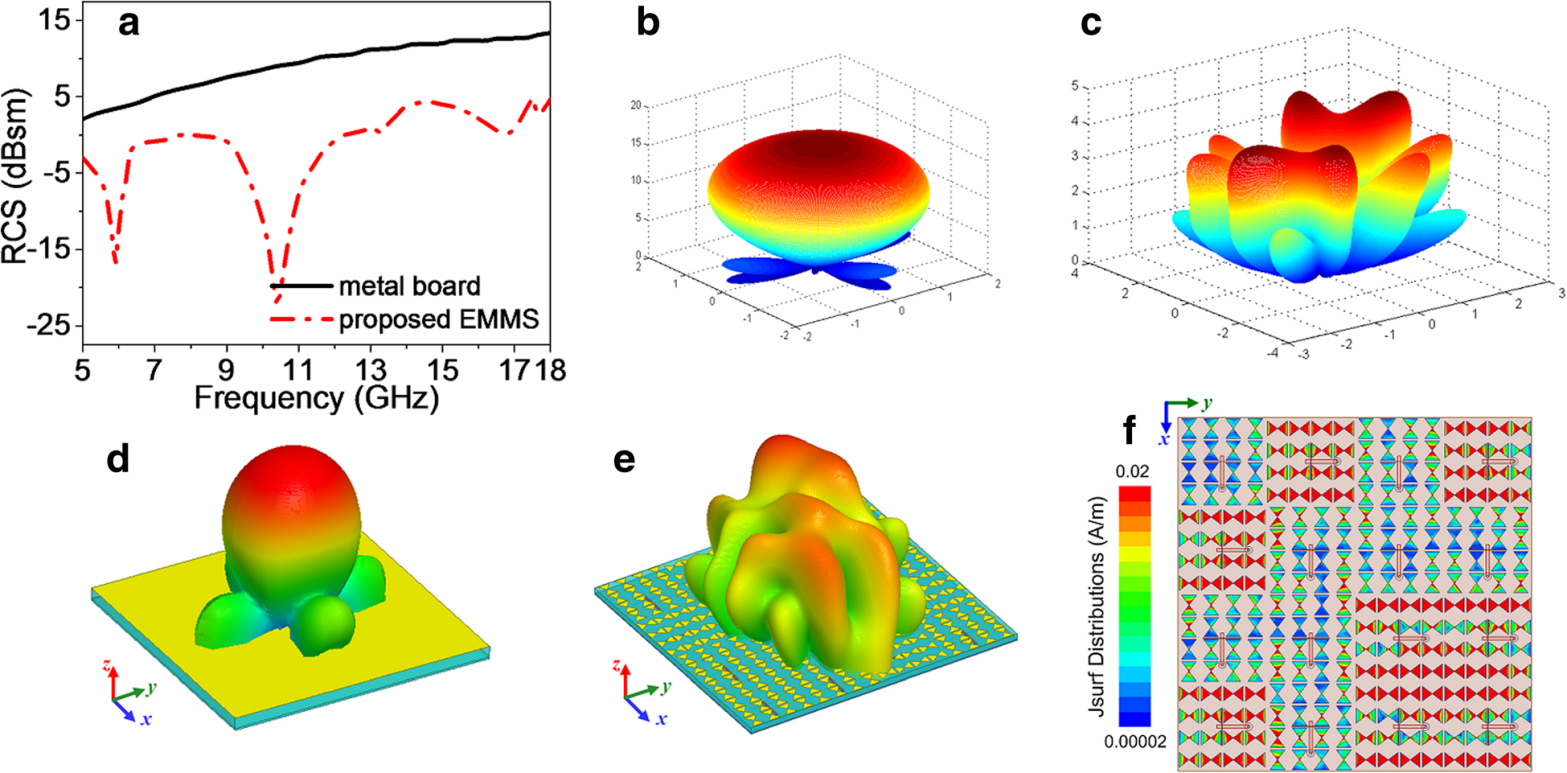
Diffusion scattering properties of the EMMS under normal incidence. **a** Radar cross section versus frequency compared with a same-sized metal board. Scattering patterns calculated by Eq. (1) for metal board (**b**) and EMMS (**c**). Scattering patterns obtained by full-wave simulations at 6 GHz for metal board (**d**) and EMMS (**e**). **f** Surface current distribution across the EMMS at 6 GHz

**Fig. 10:**
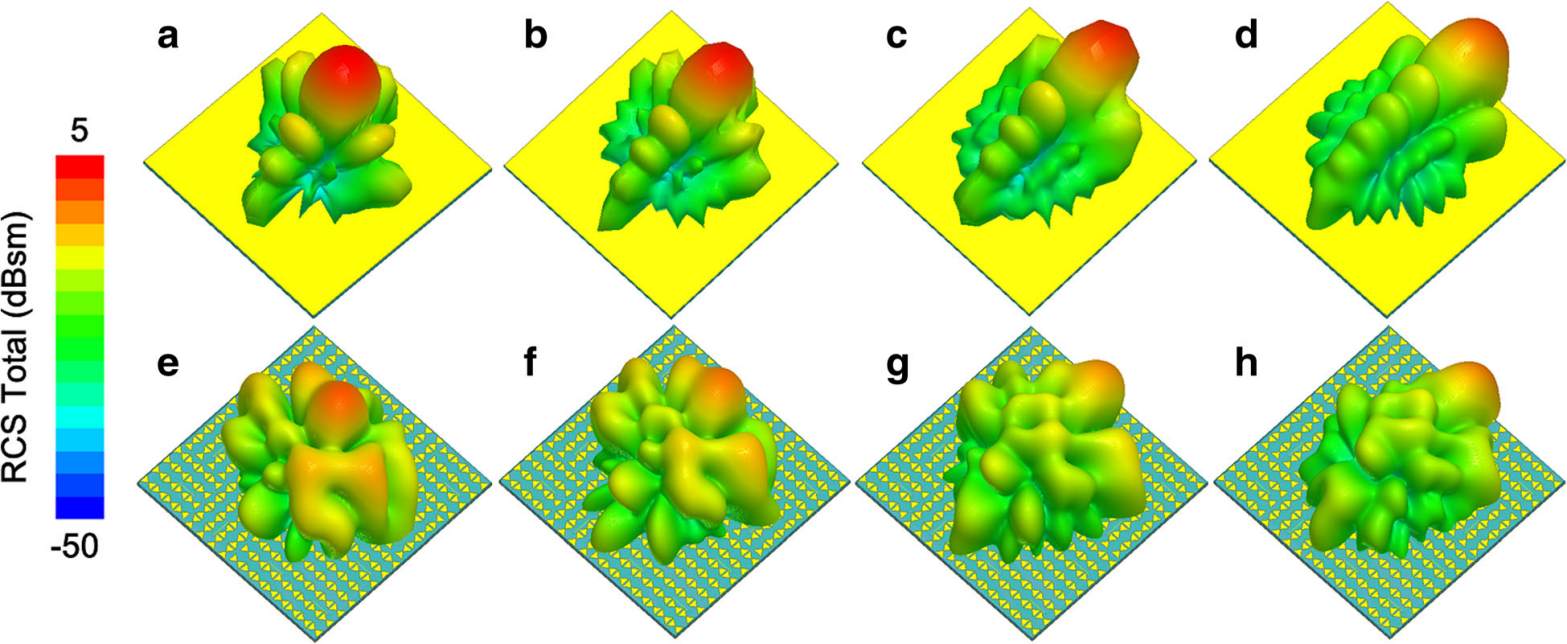
Diffusion scattering properties of the EMMS under oblique incidence at 6 GHz. **a**–**d** Scattering patterns of metal board with an incident angle of 15° (**a**), 30° (**b**), 45° (**c**), and 60° (**d**). **e**–**h** Scattering patterns of EMMS with an incident angle of 15° (**e**), 30° (**f**), 45° (**g**), and 60° (**h**)

**Fig. 11:**
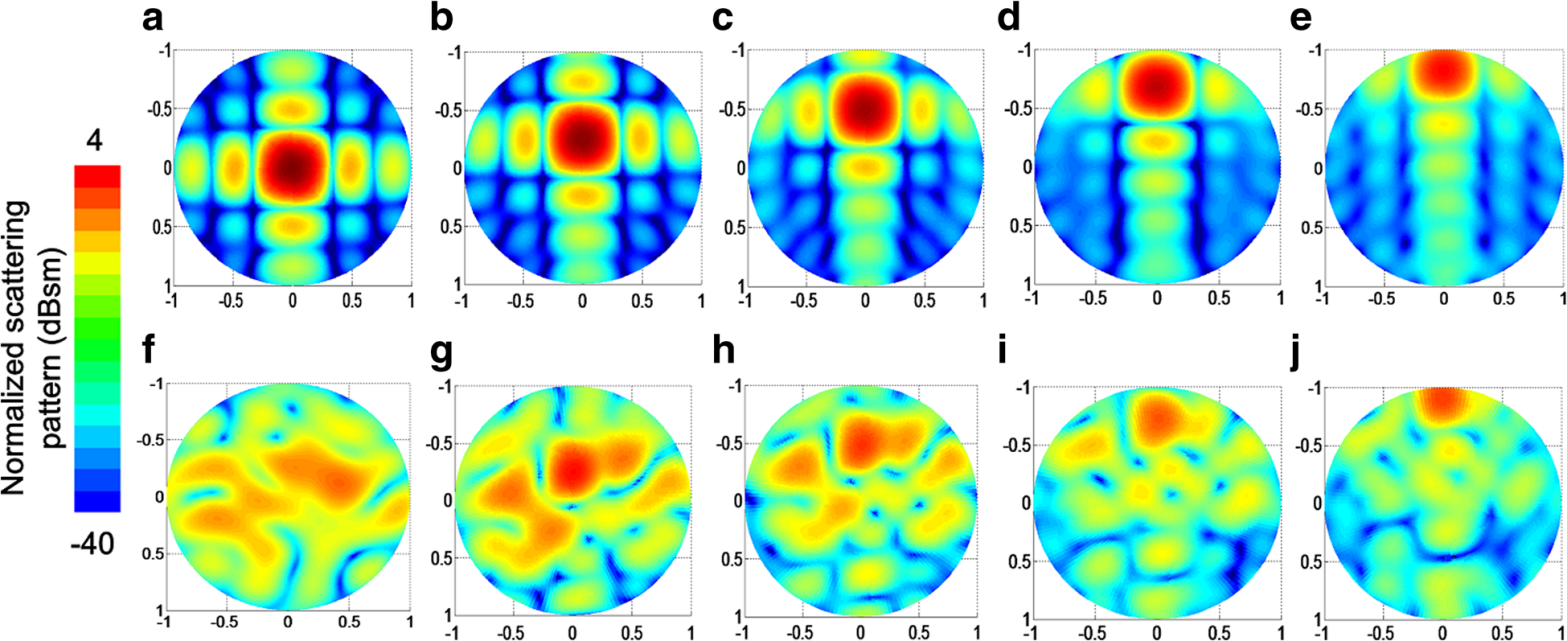
Normalized scattering patterns under oblique incidence at 6 GHz. **a**–**e** Scattering patterns of metal board with an incident angle of 0° (**a**), 15° (**b**), 30° (**c**), 45° (**d**), and 60° (**e**). **f**–**j** Scattering patterns of EMMS with an incident angle of 0° (**f**), 15° (**g**), 30° (**h**), 45° (**i**), and 60° (**j**)

For the scattering case, all of “0” and “1” elements act as passive devices. The aperiodic layout of “0” and “1” elements optimized by SAA aims at achieving diffusion scattering performance. Here, for Eq. (1), *ϕ*(*m*, *n*) represents the phase compensation of reflected wave from the (*m*, *n*) element. In terms of our proposed design, *ϕ*(*m*, *n*) evaluates 0°and 180°in correspondence to “0” and “1” elements, respectively. In order to give an intuitive demonstration of the low-scattering property of proposed EMMS, the simulated RCS result versus frequency is demonstrated compared with a same-sized metallic board. As clearly shown in Fig. 9, obvious reflection suppression is achieved in a broadband ranging from 5 GHz to 18 GHz. A continuous 6-dB RCSR is achieved nearly from 5 GHz to 18 GHz (113.04% relative bandwidth). Two RCS hollow dips appear around 5.9 GHz and 10.4 GHz with a maximum RCSR reaching up to 31.8 dB. One can tell from Fig. 9e that the scattering field of the EMMS splits into eight main small beams, which is in adequate agreement with the result obtained by the mathematical calculation in Fig. 9c. Compared with traditional chessboard configuration (four main reflected lobes), more reflected lobes contribute to each beam significantly suppressed based on energy conservation. Fig. 9f reveals the working mechanism of the EMMS. It can be observed that different elements resonate discrepantly, which yields the necessary discontinuous phase shift and finally results in diffusion reflection. The scattering properties of EMMS under oblique incidence were also investigated as shown in Fig. 10. Likewise, instead of strong specular reflection for a same-sized metal board, diffusion scattering is consecutively observed for EMMS with different incident angles. Meanwhile, as shown in Fig. 11, the normalized scattering patterns at 6 GHz with incident angles from 0° to 60° are also provided to give an intuitive demonstration of diffusion reflection. To conclude, the proposed EMMS demonstrates diffusion scattering performance in a broadband as expected.

**Fig. 12:**
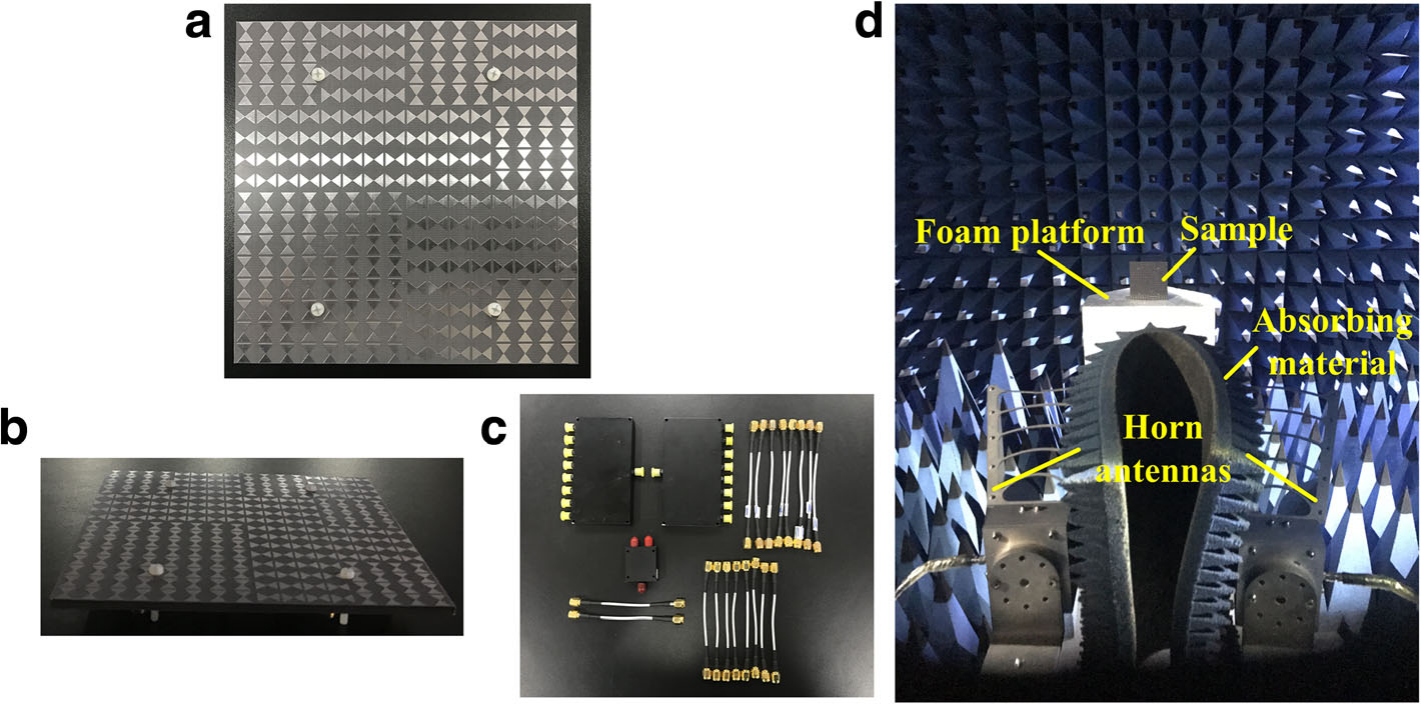
**a**, **b** Fabrication of the EMMS sample top view (**a**) and side view (**b**). **c** Power divider. **d** Basic measurement setup for scattering

**Fig. 13:**
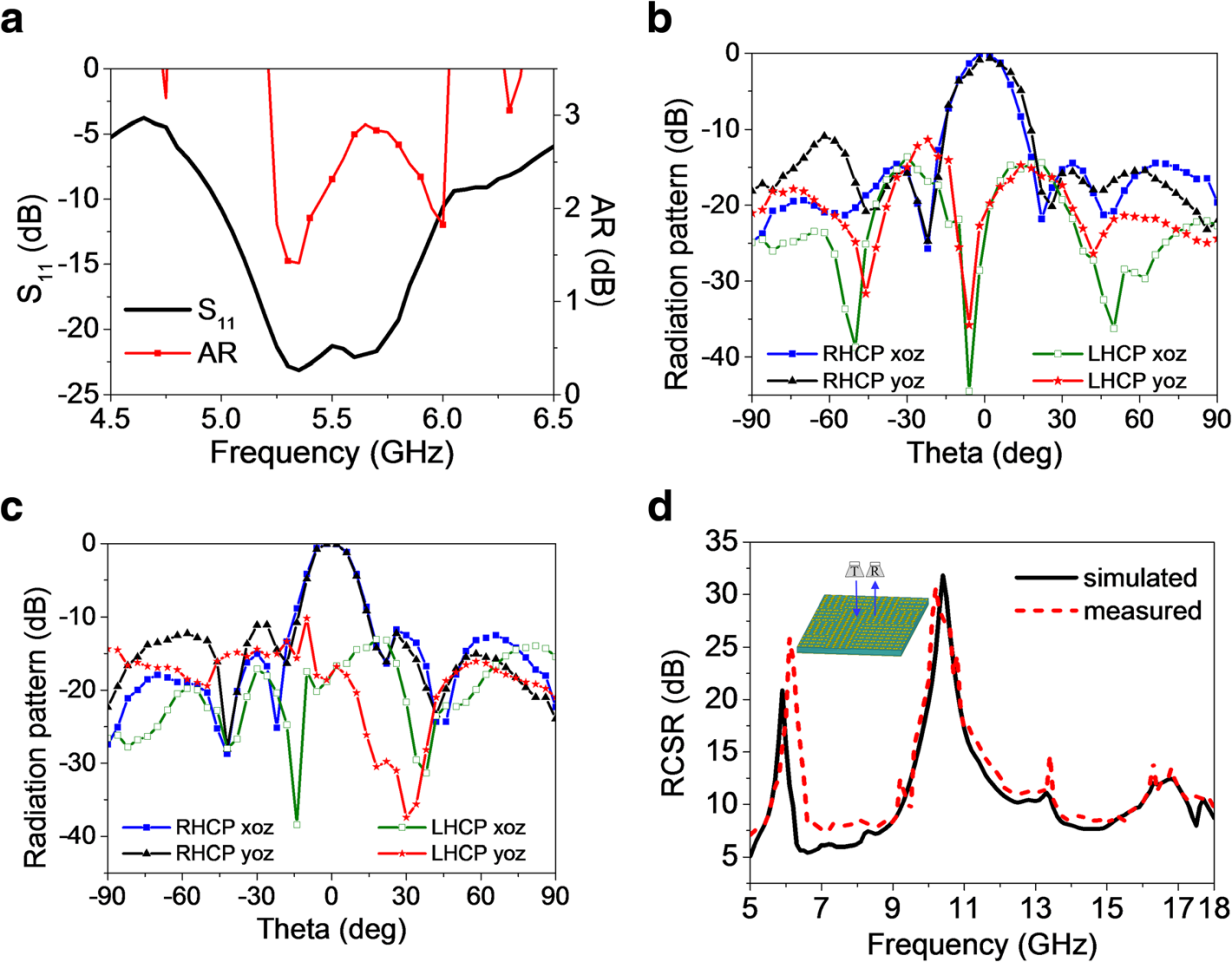
Measured radiation and scattering properties of the EMMS. **a** Measured S_11_ and AR. Normalized radiation patterns at 5.35 GHz (**b**) and 5.75 GHz (**c**). **d** Measured reflection reduction of the EMMS compared to full metal board

To validate the radiation and scattering performance mentioned above, a 4 × 4 coding EMMS sample was fabricated using standard printed circuit board (PCB) technology. The measurement was conducted in an anechoic chamber to minimize the noise interference. For the radiation case, one RS2W2080-S and two RS8W2080-S power dividers are connected in sequence to equally distribute the signal into 16 ports, while coaxial cables with different lengths are utilized to provide 90°phase shifting between “0” and “1” elements, as shown in Fig. 12. The measured bandwidths for S_11_ ≤ − 10 dB and 3 dB ARBW shown in Fig. 13a are 4.96~6.02 GHz and 5.22~6.02 GHz, respectively. The common bandwidth is from 5.22 GHz to 6.02 GHz (14.2% relative bandwidth), which is in satisfactory accordance with the simulated results. The normalized radiation patterns at 5.35 GHz and 5.75 GHz are depicted in Fig. 13b and c. Corresponding to the prediction of simulation, symmetric, normal-directed, and RHCP radiation is observed in broadside direction. The measured side lobe levels are at least 10 dB lower than the main lobe levels. In addition, the fields of RHCP are always stronger than that of LHCP by over 18.6 dB in the boresight direction. Thus, it can be concluded that the EMMS achieves good RHCP radiation performance as expected.

For the scattering case, the EMMS sample was placed vertically on the center of a foam platform, while two identical LP pyramidal horn antennas working at 1~18 GHz were placed adjacently as transmitter and receiver, respectively. A piece of absorbing material is set between the two horns to reduce undesired coupling. The centers of the sample and two horns are in the same height, and the distance between them is far enough to satisfy far-field test conditions. Gate-reflect-line calibration was also employed to further eliminate undesirable signals in the environment. The two horn antennas are connected to the two ports of VNA Agilent N5230C to evaluate reflected power on transmission coefficients. As plotted in Fig. 13d, a considerable 6-dB RCSR compared with a same-sized metal board is achieved from 5 GHz to 18 GHz (113% relative bandwidth), while over 10-dB RCSR is achieved in the band of 5.6~6.5 GHz (14.9% relative bandwidth), 9.2~13.5 GHz (37.9% relative bandwidth) and 15.9~18 GHz (12.4% relative bandwidth). Two RCSR peaks appear around 6.1 GHz and 10.2 GHz valuing 25.9 dB and 30.6 dB, respectively. The measured results agree well with the simulated ones, which verify broadband low-scattering performance of the EMMS.

Comparisons between the proposed design and former metasurface-based antenna design have been made in Table 1. In particular, [42, 45] demonstrate the performance of antenna array, while others of the single antenna. As clearly shown, the proposed EMMS yields an ultra-wideband RCSR involving in-band and out-of-band while achieving broadband tunable radiation simultaneously.

**Table 1:** Measured comparisons between the proposed design and former metasurface-based antenna design

ID	Transverse profile (mm)	Polarization mode	Operation bandwidth (GHz)	6-dB RCSR bandwidth (GHz)	Additional structure for RCSR
Ref. [41]	3.25 (0.06*λ*_0_)	LP	4.71~6.37 (29.96%)	Not investigated	–
Ref. [42]	2.3368 (0.047*λ*_0_)	LHCP	4.75–7.25 (41.67%)	Not investigated	–
Ref. [43]	3.5 (0.06*λ*_0_)	LP	4.72~6.04 (24.5%)	4.72~6.04 (24.5%)	Yes
Ref. [44]	2.4 (0.035*λ*_0_)	LHCP/RHCP/LP	4.3~4.44 for LHCP (3.2%)	4~4.65 (15%)	No
Ref. [45]	3.5 (0.058*λ*_0_)	LHCP	4.22~5.46 (25.6%)	4.53~6.7 (38.6%)	No
This paper	3.5 (0.06*λ*_0_)	LHCP/RHCP/LP	4.97~6.05 for LP (19.6%) 5.22~6 for RHCP (13.9%)	5~18 (113.04%)	No

*λ*_0_ is the free-space wavelength corresponding to the center frequency of the operation bandwidth

## Conclusions

This paper presents a novel coding EMMS with integrated broadband tunable radiation and low-scattering performance. An anisotropic element with intrinsically opposite phases under different polarized incidence is adopted as the constituent element. Appropriate feeding structures enable the anisotropic element to act as radiator. By controlling the input amplitudes and phases based on antenna array theory, LP, LHCP, or RHCP radiation can be achieved at will. In addition, the optimized layout of EMMS contributes to broadband diffusion scattering performance, which results RCSR in a broadband. Thus, broadband radiation and low-scattering performance can be simultaneously achieved in the proposed EMMS, which offers a simple, flexible, and effective strategy to solve the confliction between radiation and scattering. It is worth mentioning that the EMMS could be made up of other alternative anisotropic elements. Some application value can be expected in polarization reconfigurable antennas, target stealth, and so on.

## Abbreviations

ARBW: Axial ratio bandwidth
EM: Electromagnetic
EMMS: Electromagnetic metasurfaces
L/RHCP: Left- or right-hand circular polarization
LP: Linearly polarized
PCB: Printed circuit board
RCSR: Radar cross section reduction
SAA: Simulated annealing algorithm
